# Prevalence, Antimicrobial Resistance, and Whole Genome Sequencing Analysis of Shiga Toxin-Producing *Escherichia coli* (STEC) and Enteropathogenic *Escherichia coli* (EPEC) from Imported Foods in China during 2015–2021

**DOI:** 10.3390/toxins14020068

**Published:** 2022-01-19

**Authors:** Jinling Shen, Shuai Zhi, Dehua Guo, Yuan Jiang, Xuebin Xu, Lina Zhao, Jingzhang Lv

**Affiliations:** 1 Technology Center for Animal Plant and Food Inspection and Quarantine of Shanghai Customs, Shanghai 200135, China; jinling_zhan19@163.com (J.S.); guodehua@customs.gov.cn (D.G.); lhfzln@126.com (L.Z.); 2 School of Medicine, Ningbo University, Ningbo 315211, China; zhishuai@nbu.edu.cn; 3 Shanghai Centers for Disease Prevention and Control, Shanghai 200336, China; 4 Food Inspection and Quarantine Technology Center of Shenzhen Customs District, Shenzhen 518045, China; ficswsw@126.com

**Keywords:** Shiga toxin-producing, *Escherichia coli*, antimicrobial resistance, pathogenic potential

## Abstract

Shiga toxin-producing *Escherichia coli* (STEC) and enteropathogenic *Escherichia coli* (EPEC) are foodborne pathogens that cause hemolytic uremic syndrome and fatal infant diarrhea, respectively, but the characterization of these bacteria from imported food in China are unknown. A total of 1577 food samples from various countries during 2015–2021 were screened for STEC and EPEC, and the obtained isolates were tested for antimicrobial resistance and whole genome sequencing analysis was performed. The prevalence of STEC and EPEC was 1.01% (16/1577) and 0.51% (8/1577), respectively. Antimicrobial resistances to tetracycline (8%), chloramphenicol (8%), ampicillin (4%), ceftazidime (4%), cefotaxime (4%), and trimethoprim-sulfamethoxazole (4%) were observed. The antimicrobial resistance phenotypes corresponded with genotypes for most strains, and some resistance genes were related to mobile genetic elements. All 16 STEC isolates were *eae* negative, two solely contained *stx1* (*stx1a* or *stx1c*), 12 merely carried *stx2* (*stx2a*, *stx2d*, or *stx2e*), and two had both *stx1* and *stx2* (*stx1c* + *stx2b*, *stx1a* + *stx2a* + *stx2c*). Although they were *eae* negative, several STEC isolates carried other adherence factors, such as *iha* (5/16), *sab* (1/16), and *lpfA* (8/16), and belonged to serotypes (O130:H11, O8:H19, and O100:H30) or STs (ST297, ST360), which have caused human infections. All the eight EPEC isolates were atypical EPEC; six serotypes and seven STs were found, and clinically relevant EPEC serotypes O26:H11, O103:H2, and O145:H28 were identified. Two STEC/ETEC (enterotoxigenic *E. coli*) hybrids and one EPEC/ETEC hybrid were observed, since they harbored *sta1* and/or *stb*. The results revealed that food can act as a reservoir of STEC/EPEC with pathogenic potential, and had the potential ability to transfer antibiotic resistance and virulence genes.

## 1. Introduction

Shiga toxin-producing *E. coli* (STEC) are major foodborne pathogens, which can cause watery diarrhea, bloody diarrhea, or hemorrhagic colitis, and even life-threatening hemolytic uremic syndrome (HUS). It is estimated that STEC leads to 2,801,000 acute illnesses, 3890 HUS cases, and 230 deaths worldwide [1]. According to the U.S. Center for Disease and Control, an estimated 265,000 STEC infections, 3600 hospitalizations, and 30 deaths occur each year in the United States [2]. In China, the pathogenic *E. coli* bacteria have emerged and the human illnesses caused by these bacteria increased gradually each year from 2010 to 2019, and at present they rank 5th among all major foodborne pathogens. It is also worth noting that, among the emerging pathogenic *E. coli*, STEC infections increased most markedly [3]. STEC has been isolated from various foods, and is commonly found in retail meats in China [4,5].

The main virulence factors of STEC are Shiga toxins, which can be divided into two subfamilies, Stx1 and Stx2, encoded by *stx1* and *stx2* genes, respectively. Stx1 includes three subtypes (Stx1a, Stx1c, and Stx1d), while Stx2 contains at least seven subtypes (Stx2a, Stx2b, Stx2c, Stx2d, Stx2e, Stx2f, and Stx2g). Stx subtypes display dramatic differences in disease-causing potency. Stx2a (with or without Stx2c) and Stx2d are regarded to be more potent than other subtypes and highly associated with HUS [6]. However, the clinical significance of other Stx subtypes has also been noted [7]. The protein intimin, encoded by the *eae* gene located on the locus of enterocyte and effacement (LEE), is described in highly virulent isolates; it produces the typical “attaching and effacing” lesion on intestinal mucosa [8]. This process includes intimin, secreted effector proteins (Esp), a translocated intimin receptor (Tir), and other T3SS effectors present in the LEE island. However, strains that do not carry LEE still caused sporadic cases of HUS and other virulence factors, which are involved in alternative adherence mechanisms that may exist, such as *iha*, *saa*, *sab*, *paa*, *efa1*, *ompA*, *lpfA*, and *toxB* [9]. Other putative virulence genes, such as *ehxA*, *espP*, *etpD*, *katP*, and *subA*, usually located in a virulence plasmid were also reported [9,10]. Although O157:H7 is the most frequently reported cause of severe STEC disease and outbreaks worldwide, over the years, more than 400 STEC serotypes were found to be associated with human illnesses, including the notably “top 6” non-O157 STEC serogroups (O26, O45, O103, O111, O121, and O145) that have emerged as rising human enteric pathogens responsible for outbreaks and sporadic cases of illnesses worldwide [9].

Enteropathogenic *E. coli* (EPEC) can cause infant fatal diarrhea mainly in developing countries, and are isolated from various kinds of food and animals. Previous studies demonstrated that in China, EPEC is one of the most common diarrheagenic *E. coli* [11,12] and were found in many food sources (i.e., ready-to-eat food, poultry, pork, and beef) [13,14]. EPEC is a group of *E. coli* strains that are positive for *eae* but negative for *stx*. In addition to *eae*, typical EPEC (tEPEC) strains carried the EPEC adherence factor (pEAF) plasmid that encodes the bundle forming pili (BFP), while atypical EPEC (aEPEC) do not possess this pEAF. A number of genes have been identified as being of particular importance for the pathogenicity of EPEC, including the *eae* gene; *espA*-*B* (encoding type III secretion system proteins); *tir* (encoding the translocated intimin receptor), *katP* (encoding a bifunctional catalase peroxidase); *etpD* (associated with a type III secretion system); *toxB* (the adherence-associated gene); *iha* (encoding the IrgA homologue adhesin); and *saa* (encoding an auto agglutinating adhesin) [15,16,17].

The pathogenicity of STEC and EPEC is complex, thus the virulence potential should be assessed not only based on serotypes, but also other virulence associated markers, for instance, virulence genes, virulence gene subtypes, and phylogenetic markers. Whole genome sequencing (WGS) reveals the entire spectrum of pathogen information; moreover, the phylogenetic relationship between strains from different sources and geographic regions can be deduced by WGS.

The increase in antimicrobial-resistant *E. coli* has become a major public health threat all over the world [18]. Antimicrobial resistant strains may be transferred to humans through the consumption of contaminated food. Additionally, the antimicrobial resistance genes can be transferred among normal intestinal flora, thus bringing challenge to infection treatment.

With the accelerating globalization of food trade, STEC and EEPC can be transmitted all over the world through contaminated food. Until recently, there has been no report on the characterization of STEC and EPEC from imported food in China. Therefore, we isolated these bacteria from food imported from different continents, and performed the antimicrobial resistance and whole genome sequencing analysis, so as to provide technical support for food safety investigations and monitor the emergence of *E. coli* in foods.

## 2. Results

### 2.1. Prevalence of STEC and EPEC

Of the 1577 imported food samples from 2015 to 2021, 34 (2.16%) and 41 (2.60%) were tested positive for *stx* and *eae*, respectively. A total of 16 (1.01%) food samples were confirmed to be contaminated with STEC, and 8 (0.51%) food samples contaminated with EPEC (Table 1). The STEC/EPEC prevalence in different imported food samples (i.e., frozen beef, frozen pork, and frozen mutton) are shown in Table 1.

### 2.2. Antimicrobial Resistance Phenotype

**Figure 1:**
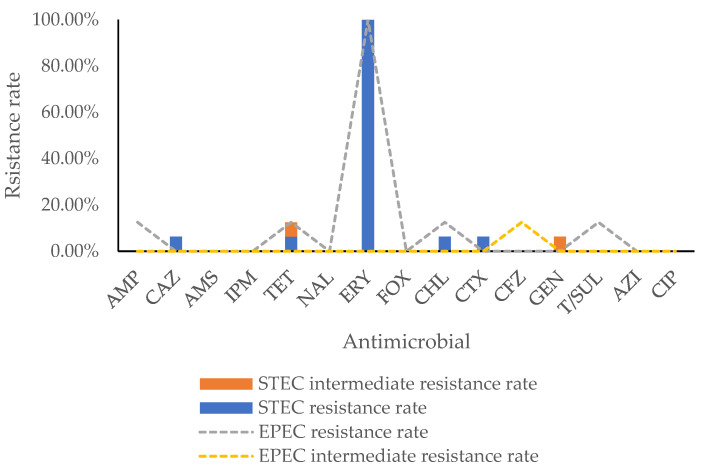
Resistance rates of STEC and EPEC isolates to 15 antimicrobials.

The antimicrobial resistance of 16 STEC and 8 EPEC strains is presented in Figure 1. All isolates were naturally resistant to erythromycin; therefore, erythromycin was excluded from the following analysis. A total of four isolates were found resistant. Generally, the highest resistance rates (8.33%, 2/24) were observed for tetracycline and chloramphenicol, followed by ampicillin (4.17%, 1/24), ceftazidime (4.17%, 1/24), cefotaxime (4.17%, 1/24), and trimethoprim-sulfamethoxazole (4.17%, 1/24). Concerning the STEC isolates, resistances were only observed for ceftazidime (6.25%, 1/16), tetracycline (6.25%, 1/16), chloramphenicol (6.25%, 1/16), and cefotaxime (6.25%, 1/16), and intermediate resistances to tetracycline (6.25%, 1/16) and gentamicin (6.25%, 1/16) were also found. Regarding EPEC, 12.50% (1/8) of the isolates were resistant to ampicillin, tetracycline, chloramphenicol, and trimethoprim-sulfamethoxazole, respectively, and 12.5% of the isolates were intermediate resistant to cefazolin (12.50%, 1/8). Regarding multiple antimicrobial resistance, four isolates (16.7%, 4/24) were found to be resistant to two antibiotics, and two of them were intermediate to another antibiotic.

AMP, ampicillin; CAZ, ceftazidime; AMS, ampicillin-sulbactam; IPM, imipenem; TET, tetracycline; NAL, nalidixic acid; ERY, erythromycin; FOX, cefoxitin; CHL, chloramphenicol; CTX, cefotaxime; CFZ, cefazolin; GEN, gentamicin; T/SUL, trimethoprim-sulfamethoxazole; AZI, azithromycin; and CIP, ciprofloxacin.

### 2.3. Antimicrobial Resistance Genotype

All the antimicrobial resistant strains carried the respective resistance genes, except for one strain (816b) that showed resistance to ceftazidime and cefotaxime, while no resistance genes, such as the extended spectrum β-lactamase genes, were found (Table 2). Two tetracycline-resistant strains (1053l-2, 1509-1) were found to harbor tetracycline-resistant genes, *tetA* + *tetB* and *tetA*, respectively; two chloramphenicol-resistant strains (1053l-2, 1095a) carried chloramphenicol-resistant genes, *catA1* and *cmlA1*, respectively; one ampicillin-resistant isolate (1509-1) carried the *blaTEM-1B* gene, which encodes resistances to β-lactams and is linked to extended spectrum β-lactamases (ESBLs) production, thus this isolate also showed intermediate resistance to cefazolin; one trimethoprim-sulfamethoxazole-resistant isolate carried the respective *sul3* and *dfrA12* genes; and one gentamicin-resistant isolate carried multiple gentamicin-resistant genes, including *aadA1*, *aph*(*4*)*-Ia*, *aac*(*3*)*-IV*, *aph*(*6*)*-Id*, and *aph*(*3″*)*-Ib*.

For the majority of the time, the strains carrying antimicrobial resistance genes showed the respective resistance/intermediate resistance phenotype (Table 2). There was one strain harboring the sulfonamide resistance gene, *sul1*, but did not show resistance or intermediate resistance to trimethoprim-sulfamethoxazole. The strain 1509-1 carried the ciprofloxacin resistance gene *qnrS1*, thus the ciprofloxacin MIC reached 0.25, while for other strains it was 0.03 (Table 2).

The multiple antimicrobial resistant strains also had multiple resistance genes. The location of these resistance genes was evaluated. For 1053l-2, the *aadA1*, *sul1*, and *qacE* were found on the same contig 181; *aph*(*3′*)*-Ib* and *aph*(*6*)*-Id* were on contig 321; *aac*(*3*)*-IV* and *aph*(*4*)*-Ia* were on contig 279; the co-occurrence of *aadA1*, *cmlA1*, and *sul3* on contig 102; *aadA8b*, *aadA2*, and *dfrA12* on contig 103 were found for 1095a; and for strain 1509-1, the co-occurrence of *qnrS1* and *blaTEM-1B* were observed on contig 54. Furthermore, the relations of these antimicrobial resistance genes to mobile genetic elements were evaluated. The *aac*(*3*)*-IV* and *aph*(*4*)*-Ia* were associated with insertion sequence ISEc59. The gene *blaTEM-1B* was related to insertion sequence ISKpn19 while *qnrS1* was related to unit transposon Tn2.

### 2.4. Virulence Gene Analysis

Among the 16 STEC strains, two solely contained *stx1* (*stx1a* or *stx1c*), 12 merely carried *stx2* (*stx2a*, *stx2d*, *stx2e*), and two had both *stx1* and *stx2* (*stx1c* + *stx2b*, *stx1a* + *stx2a* + *stx2c*) (Table 3). All the STEC isolates were *eae* negative; however, other adherence factors, such as *iha* (5/16), *sab* (1/16), and *lpfA* (8/16), were found. Six isolates carried the hemolytic gene *ehxA* (25%, 6/16), and all belonged to phylogroup B1. The most widespread genes that were detected in nearly all the STEC isolates were *terC* (tellurium ion resistance protein), *traT* (outer membrane protein complement resistance), *iss* (increased serum survival), and *ompT* (outer membrane protease). Some isolates carried toxins, for instance, the sublitase toxin *subA* (4/16), EAEC heat-stable enterotoxin I *astA* (6.25%, 1/16), colicin ia (*cia*) (7/16), colicin B (*cba*) (3/16), colicin M (*cma*) (4/16), colicin ib (*cib*) (4/16), colicin E1 (*cea*) (4/16), and colicin H (*mchB*) (1/16). Some isolates had the enteroaggregative immunoglobulin repeat protein encoding genes, *air* (4/16) and *epeA* (4/16), and the plasmid-encoded extracellular serine protease gene *espP* (4/16). One STEC isolate had ETEC specific genes *sta1* (heat-stabile enterotoxin ST-Ia) and *stb* (heat-stabile enterotoxin II), and the other one carried *sta1*.

None of the eight EPEC isolates had EAF, and thus were aEPEC. All the EPEC isolates had the following genes, including *terC*, *tir*, *espF*, *espA*, and *nleB*. More than half the EPEC isolates harbored *ehxA* (5/8), *ompT* (5/8), *iss* (6/8), *traT* (7/8), *gad* (6/8), *espB* (6/8), *nleA* (5/8), *tccP* (5/8), *espJ* (4/8), *etpD* (4/8), and *nleC* (4/8). Apart from *eae*, some isolates also had other adherence associated genes, including *afaD* (2/8), *efa1* (1/8), and *iha* (1/8). One isolate also had the ETEC-specific gene *sta1*.

### 2.5. Serotype, ST, and Phylogenetic Analysis

**Figure 2:**
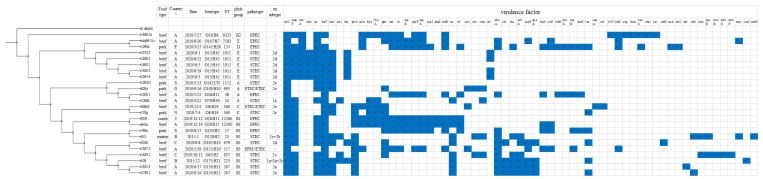
Serotype, ST, phylogroup, virulence factor, and phylogenetic analysis of the STEC and EPEC isolates in this study. 1 A, Australia; F, France; S, Spain; G, German; N, the Netherlands; I, Indonesia; B, uncertain; C, Argentina.

Among the 16 STEC isolates, 10 serotypes were found: O142: UN, O8:H19, O79:H40, O15:H45, O163:H19, O130:H11, O100:H30, O43:H2, O175:H21, and O128:H2. Ten STs were identified: ST1112, ST360, ST10, ST1011, ST679, ST297, ST993, ST937, ST223, and ST25. The serotypes corresponded with the STs, and five, two, and two and one serotypes (STs) belonged to phylogroups B1, A, E, and C, respectively (Figure 2).

Regarding the 8 EPEC isolates, 6 serotypes were observed: O26:H11, O145:H28, O103:H2, O56:H6, O107:H7, and O153:H19, and 7 STs were found, including ST137, ST17, ST48, ST6323, ST7583, ST517, and one newly identified ST12360 named by EnteroBase. Serotype was highly correlated with ST, except for O26:H11, which belonged to different STs. Three, one, one, one, and one STs belonged to phylogroups B1, B2, E, D, and A, respectively (Figure 2).

The phylogenetic tree based on cgMLST showed that the strains in this study were diverse. Regardless of the pathotype (STEC/EPEC), serotype, or ST, all the strains were grouped according to their phylogroups, as isolates belonging to different phylogroups (B2, D/E, A, C, and B1) clustered together (Figure 2). Some virulence genes were previously found in phylogroup B1, for instance, *lpfA*, *ehxA*, *iha*, *subA*, *espP*, and *epeA*. Certain *stx* subtypes were found to be associated with food type or serotype. For instance, *stx2e* was mainly found in pork, *stx2a* and *stx2d* were more commonly found in beef. Genotype *stx1c* + *stx2b* was observed in O128:H2 from mutton. There was no apparent correlation between country, food type, serotype, and ST; however, five isolates from beef in country A showed highly similar characteristics, regarding serotype, ST, and virulence genes.

## 3. Discussion

The characterization of STEC and EPEC from imported food in China has not been reported previously. Therefore, in this study, we isolated these bacteria from various kinds of food, mainly animal source frozen meat, imported from different countries, and studied their antimicrobial resistance, genetic diversity, and virulence profile.

The overall PCR screening rates for STEC (2.16%) and EPEC (2.60%) and the isolation rates for STEC (1.01%) and EPEC (0.51%) in this study were both lower than in the previous reports [4,19,20,21]. For instance, the PCR screening rates for STEC were 19.5% for locally produced retail raw meats in China [4], 49.3% for ground beef in Chile [19], 8.5% for ground beef and 13.4% for ground pork in the United States [20], and 8.4% for fresh beef in Italy [21]; the STEC isolation rates were 6.8% for retail raw meats in China [4], 10% for ground beef in Chile [19], 5.2% for both ground beef and ground pork in the United States [20], 3.7% for fresh beef in Italy [21], and 2% for cow’s milk in Spain [18]. EPEC was detected in 8.5% of ready-to-eat samples in China [13] and in 6% of cow’s milk in Spain [18]. The food samples in this study were mainly frozen meat, which is not suitable for STEC/EPEC survival or multiplication. This may be one reason for the low STEC/EPEC prevalence in this study. In addition, good hygiene control measures may be taken by the overseas food manufactures. Furthermore, different enrichment and isolation methods may be used by different studies. Nevertheless, similar to previous studies, the STEC isolation rates were lower than PCR screening rates. The interfering high levels of background microflora, the presence of other bacteria carrying *stx*, the low levels of STEC in the samples, or the presence of free Stx phages can lead to the failure of STEC isolation [22]. Currently, there is no suitable selective isolation agar for STEC, therefore the isolation of the suspected STEC colonies from selective/differential agar media is challenging. Most STEC isolates in this study were obtained from MacConkey and TBX, not from Chromagar^TM^ STEC media, which has inhibitory effects on uncommon non-O157 serotypes [23,24]. Therefore, we recommend using Chromagar™ STEC together with *E. coli* differentiating agar, e.g., TBX or MacConkey, to facilitate the isolation.

Drug resistance in *E. coli* has become a worldwide issue. Overall, the antimicrobial resistance is not severe in the studied isolates. Resistance to β-lactam, tetracycline, chloramphenicol, and sulfonamide are most frequently detected in this study, quite different from the situation in China, for which streptomycin (46.94%) and ciprofloxacin (20.41%) resistances were most common for the STEC from retail meats [5]. It is worth noting that some multi-drug resistant isolates carrying multiple antimicrobial resistance genes on the same contigs were found in this study. The co-occurrence of multiple antibiotic resistance genes could show the extensive administration of antimicrobials over many years and it may have led to the development of multiple resistances by mobile genetic elements, resulting in co-selection. The mobile genetic element-associated resistance genes were also found in this study, and this poses a great challenge to the combat against bacterial antimicrobial resistance, since they can easily be horizontally transferred among bacteria. The antimicrobial resistance phenotypes are in accordance with the genotypes for most antimicrobials; however, there is a mismatch between them in a few isolates, a finding that has also been reported in other species [25,26]. The observed phenotypic resistance strains lacking resistance genes might be due to non-resistance genetic factors, while resistance genes detected in susceptible isolates might be considered as “silent” or unexpressed genes [26].

The virulence potential of STEC should be assessed based on various factors, including serotype, *stx* subtype, virulence gene, phylogroup, and ST. We detected several STEC serotypes that have been related to human infections, such as O8:H19, O128:H2, O100:H30, and O130:H11, based on previous reports [27] and the EnteroBase database. Certain subtypes of the *stx2* subtypes (*stx2a*, *stx2c*, and *stx2d*) were reported to be linked with serious human diseases [6]. Nine isolates in this study possessed the above subtypes, thus posing a health threat. Stx2e-producing STEC strains have also been isolated from patients with acute diarrhea and HUS, thus the clinical significance of the four isolates carrying *stx2e* in this study should not be neglected. Specifically, the two *stx2e*-positive O8:H19-phylogroup C isolates belonged to ST360, which is principally found in human diseases based on the EnteroBase database, and possessed adhesion and colonization factor *lpfA*; thus, they should also be attached importance. Strains belonging to *E. coli* phylogroups B1, C, and E2(O157) are often pathogenic and of interest to medical research [28]. We found that some genes were highly associated with phylogroup B1, such as *lpfA*, *ehxA*, *iha*, *subA*, *espP*, and *epeA*. Strains positive for these virulence genes may have a pathogenic potential. Iha is an adherence conferring protein and a siderophore receptor distributed among STEC, and it may be involved as an alternative mechanism of adhesin in *eae*-negative STEC strains [29]. Subtilase cytotoxin, encoded by the *subA* and *subB* genes, is harbored by the O113:H21 outbreak strain and other *eae-*negative strains associated with human diseases [30]. Strains harboring *stx2a* and *eae*/*aggR* were assessed to be on the highest level for their estimated potential to cause diarrhea, bloody diarrhea, and HUS [9]. We did not detect the virulence combination in the studied isolates. However, the two O130:H11 isolates within phylogroup B1 possessing *stx2a* + *iha* + *subA* + *ehxA* + *lpfA* may have a high pathogenic potential.

None of the isolates obtained in this study were typical EPEC, which is in line with the previous results on various kinds of food [13,31]. The aEPEC strains are considered as emerging entero-pathogens detected worldwide [18]. Recent studies indicated that typical EPEC cases of diarrhea have been replaced with atypical EPEC in both developing and industrialized countries [32]. The *stx* genes are carried by lambdoid phages, which are highly mobile genetic elements, and the horizontal transfer and the dissemination, as well as the loss of the *stx* genes, are facilitated [33]. The aEPEC can also include EHEC and EPEC that have lost the *stx* genes and *bfp* genes during passage through a host or the environment or after culture in the laboratory; thus, these bacteria should not be underestimated. Clinically relevant serotypes O26:H11 and O103:H2 were found for two EPEC isolates in phylogroup B1, thus posing a great health threat. Although sharing the same serotype, O26:H11, the virulome was quite different for those belonging to B1 (12360) and A (ST48), indicating that their virulence potential may be distinct.

Notably, we found two STEC and one EPEC carrying *sta1* and/or *stb*, which were the virulence markers of enterotoxigenic *Escherichia coli* (ETEC), and thus were STEC/ETEC and EPEC/ETEC hybrids, respectively. The *E. coli* genome is dramatically plastic and this accelerates the adaptation of this species into various environments, which provides numerous opportunities for new variants to emerge via the gains and losses of genes. Recently, hybrid *E. coli* pathotypes are representing emerging public health threats with enhanced virulence from different pathotypes. The most notorious hybrid was the STEC/EAEC strain O104:H4, which caused a large outbreak with numerous HUS cases and deaths in Germany in 2011 [34]. STEC/ETEC hybrids have been reported to be associated with diarrheal disease and hemolytic uremic syndrome (HUS) in humans [35,36], and EPEC/ETEC hybrid types have also been reported from patients [37]. The horizontal transmission of *stx* and/or *sta/stb* genes by the independent acquisition of the Stx-phages and/or plasmids carrying these genes lead to the emergence of STEC/ETEC hybrids. The STEC/ETEC hybrid (826a) identified in this study had the same virulence characteristics as the STEC/ETEC strain causing diarrhea in an 82-year-old patient in Sweden. For instance, both strains belonged to O100:H30, ST993, and phylogroup A and carried *stx2e* + *sta1*; therefore, it also had a high potential to cause human diseases [38]. It is also worth noting that the STEC/ETEC hybrid 908e2 possessing two *st* variants, *sta1* and *stb*, had a distinctly mucoid and thread-drawing morphology on the nutrient agar. The genetic mechanism should be further explored.

## 4. Conclusions

The contamination frequency of STEC and EPEC from imported foods during the period study was relatively low, and the antimicrobial resistance was not severe. However, multidrug-resistant isolates harboring the respective multiple antimicrobial resistance genes, which are related to mobile genetic elements, were identified, and thus have the potential to transfer antibiotic resistance. Some isolates carried the virulence factors described in pathogenic strains and thus have a high pathogenic potential. STEC/ETEC and EPEC/ETEC hybrid strains were identified. Since the virulence genes of *E. coli* are usually located on plasmids or prophages, the emerging *E. coli* hybrids in foods may have enhanced virulence and thus should be attached with great importance.

## 5. Materials and Methods

### 5.1. Sample Collection

A total of 1577 samples from different kinds of foods imported from different continents were collected from the containers or airplanes at Shanghai port from 2015 to 2021, including frozen beef (*n* = 1066), frozen pork (*n* = 172), fresh and frozen aquatic products (*n* = 198), frozen mutton (*n* = 102), milk products (*n* = 27) and frozen chicken (*n* = 12). The samples were collected according to the National Sampling Plan by authorities and sent directly to the laboratory for testing.

### 5.2. Strain Isolation and Identification

The enrichment method was based on the ISO/TS 13136:2012 [39]. Briefly, a 25 g portion of each sample was transferred into a sterile sample filter bag containing 225 mL of sterile modified tryptone-soya broth (mTSB) (Land Bridge, Beijing, China), then incubated at 37 °C for 18–24 h. After enrichment, 1 mL of culture was centrifuged and performed for DNA extraction by using the bacterial genomic DNA extraction kit (TIANGEN biotech co., Beijing, China), following the manufacturer’s instructions. DNA was screened for the presence of *stx* and *eae* according to the method of USDA FSIS MLG-5 [40]. The 25 μL reaction mixture contained 12.5 μL of real-time PCR premix (Takara, Dalian, China), 1.26 μM of *stx* (*stx1* and *stx2*) primers, 1 μM of *eae* primers, 0.16 μM of 16 s primers, 0.25 μM of *stx1* probe, 0.25 μM of *stx2* probe, 0.2 μM of *eae* probe, 0.1 μM of 16 s probe, and 5 μL of DNA template. The PCR reaction conditions were as follows: 95 °C pre-denaturation for 2 min, followed by 40 cycles of 95 °C denaturation (5 s) and 60 °C (34 s) annealing. The EHEC O157: H7 strain EDL933 and nucleotide-free water were included as the positive and blank controls, respectively. For the *stx* positive samples, the enrichment was streaked onto three solid media: CHROMagar™ STEC agar (CHROMagar, Paris, France), MacConkey agar (Land Bridge, Beijing, China), and TBX agar (Oxoid, UK); for the *eae* positive samples, the enrichment was streaked onto two solid media: MacConkey agar (Land Bridge, Beijing, China) and TBX agar (Oxoid, UK). Approximately 40 colonies with *E. coli* morphology were picked from the above agars for further *stx* or/and *eae* detection. Each *stx*/*eae*-positive isolate was confirmed to be *E. coli* by the API 20 E system (bioM’erieux, Lyon, France).

### 5.3. Antimicrobial Susceptibility Test

The antimicrobial resistance patterns of the 24 isolates were determined by the micro-dilution method, following the standards of the Clinical and Laboratory Standards Institute [41]. The following 15 antibiotics were tested using the commercial 96-well antibiotic plate (Meihua Medical Tech co., Zhuhai, China): ampicillin (AMP); ceftazidime (CAZ); ampicillin-sulbactam (AMS); imipenem (IPM); tetracycline (TET); nalidixic acid (NAL); erythromycin (ERY); cefoxitin (FOX); chloramphenicol (CHL); cefotaxime (CTX); cefazolin (CFZ); gentamicin (GEN); trimethoprim-sulfamethoxazole (T/SUL); azithromycin (AZI); and ciprofloxacin (CIP). The breakpoints for resistance, intermediate and susceptible, were referred to CLSI [41].

### 5.4. Whole Genome Sequencing Analysis

#### 5.4.1. DNA Extraction

Genomic DNA from each strain was extracted from overnight cultures using the bacterial genomic DNA extraction kit (TIANGEN biotech co., Beijing, China), following the manufacturer’s instructions. The DNA concentration was determined using the Qubit™ dsDNA HS Assay Kit (Thermo Fisher Scientific, Waltham, MA, USA). The DNA integrity was determined by 1% gel electrophoresis. The qualified DNA was stored in −20 °C until use.

#### 5.4.2. Whole Genome Sequencing and Contig Assembly

The library was constructed using NEB Next^®^ Ultra™ DNA Library Prep Kit for Illumina (NEB, England). The genomes of the strains sequenced used an Illumina HiSeq sequencer (Illumina, San Diego, CA, USA), with the 2 × 150 bp pair-end chemistry according to the manufacturer’s instructions, at approximately 350X average coverage. The sequencing reads were quality-control processed and quality evaluated with FastQC (http://www.bioinformatics.babraham.ac.uk/projects/fastqc/, accessed on 21 October 2021). The processed reads were assembled de novo with SPAdes (version: 3.12.0) in “careful mode”.

#### 5.4.3. Molecular Characterization of the Strains Based on WGS

The serotype of each strain was determined using the genes deposited in the Center for Genomic Epidemiology (http://www.genomicepidemiology.org, accessed on 21 October 2021) for *E. coli* as part of their web-based serotyping tool (SerotypeFinder 2.0—https://cge.cbs.dtu.dk/services/SerotypeFinder/, accessed on 21 October 2021), with a similarity of 85% and minimum length of 60%; the ST of each strain was in silico analyzed using the MLST *E. coli*#1 approach (*dnaE*, *gyrB*, *recA*, *dtdS*, *pntA*, *pyrC*, and *tnaA*) provided by the Center for Genomic Epidemiology (https://cge.cbs.dtu.dk/services/MLST/, accessed on 21 October 2021); and the phylogroup was determined by the using the *E.coli* phylotype analysis available in the EnteroBase website (https://enterobase.warwick.ac.uk/species/ecoli/search_strains, accessed on 21 October 2021). The virulence genes present in each strain were determined using the genes deposited in the Center for Genomic Epidemiology for *E. coli* as part of their web-based VirulenceFinder 2.0 tool (https://cge.cbs.dtu.dk/services/VirulenceFinder/, accessed on 21 October 2021), with the similarity of 90% and minimum length of 60%; the resistance genes present in each strain were identified using the genes deposited in the Center for Genomic Epidemiology for *E. coli* as part of their ResFinder 4.1 tool (https://cge.cbs.dtu.dk/services/ResFinder/, accessed on 21 October 2021), with a similarity of 90% and minimum length of 60%. The relations of the antimicrobial resistance genes to the mobile genetic elements were evaluated using the mobile element finder v1.0.3 tool provided by the Center for Genomic Epidemiology (https://cge.cbs.dtu.dk/services/MobileElementFinder/, accessed on 30 November 2021).

#### 5.4.4. Phylogenetic Analysis

The phylogenetic relationship of the strains was assessed by a core genome multilocus sequence typing (cgMLST) analysis. The core genome of all the *E. coli* strains was described using REALPHY 1.12 [42]. Specifically, one of the *E. coli* strains was randomly selected as reference, and the genome sequences of all the other strains were then mapped to the reference genome to identify their core genome. A core genome phylogenetic tree was constructed using RAxML Version 8.2.4 with GTRGAMMA option and *E. albertii* was used as the outgroup [43]. The phylogenetic tree was visualized using the Interactive Tree of Life (iTOL) [44,45].

#### 5.4.5. Nucleotide Sequence Accession Numbers

The draft genome sequences of the *E. coli* strains used in this study are available in GenBank under the following accession numbers: JAIPMO000000000; JAJIYZ000000000; JAIPMP000000000; JAIPMQ000000000; JAJIZA000000000; JAJIZB000000000; JAJIZC000000000; JAIPMR000000000; JAIPMT000000000; JAJIZD000000000; JAIPMU000000000; JAJIZE000000000; JAJIZF000000000; JAIPMV000000000; JAIPMW000000000; JAIPMX000000000; JAIPMY000000000; JAIPMZ000000000; JAIPNA000000000; JAJIZG000000000; JAIPNB000000000; JAJIZH000000000; JAJTCC000000000; and JAJTCB000000000.

## Figures and Tables

**Table 1:** Prevalence of STEC and EPEC in imported food samples.

Food Type (*n*)	STEC Prevalence (%)	EPEC Prevalence (%)
Frozen beef (*n* = 1066)	12 (1.13%)	5 (0.47%)
Frozen pork (*n* = 172)	3 (1.74%)	2 (1.16%)
Frozen and fresh aquatic products (*n* = 198)	0	1 (0.51%)
Frozen mutton (*n* = 102)	1 (0.98%)	0
Milk products (*n* = 27)	0	0
Frozen chicken (*n* = 12)	0	0
Total (*n* = 1577)	16 (1.01%)	8 (0.51%)

**Table 2:** Antimicrobial resistance phenotype and genotype for the resistant/intermediate resistant isolates in this study.

Strain ID	Food Type	Antimicrobial Resistance Phenotype		Antimicrobial Resistance Genotype
		Resistance	Intermediate	
1053l-2	Pork	Tetracycline + chloramphenicol	Gentamicin	*tet*(*A*)*, tet*(*B*)*, catA1, mph*(*B*)*, aadA1, aph* (*4*)*-Ia* ^1^*, aac*(*3*)*-IV* ^1^*, aph*(*6*)*-Id, aph*(*3″*)*-Ib, sul1, qacE, mdf*(*A*) ^5^
1095a	Pork	Chloramphenicol + trimethoprim-sulfamethoxazole		*aadA1, aadA8b, aadA2, cmlA1, sul3, dfrA12, mdf*(*A*) ^5^
1509-1	Beef	Ampicillin + tetracycline	Cefazolin	*blaTEM-1B*^2^*, tet*(*A*)*, qnrS*^3,4^*, mdf*(*A*) ^5^
816b	Beef	Ceftazidime + cefotaxime		*mdf*(*A*) ^5^
1789-1	Beef		Tetracycline	*mdf*(*A*) ^5^

^1^ Insertion sequence, ISEc59-related. ^2^ Insertion sequence, ISKpn19-related. ^3^ Unit transposon, Tn2-related. ^4^ The ciprofloxacin MIC for 1509-1 reached 0.25, compared with 0.03 for other strains in this study, though it did not show resistance to ciprofloxacin. ^5^ All *E. coli* were naturally resistant to erythromycin and carried *mdf*(*A*).

**Table 3:** The number of STEC and EPEC isolates positive for respective virulence genes (VGs).

VG	STEC	EPEC	VG	STEC	EPEC	VG	STEC	EPEC	VG	STEC	EPEC
*stx1a*	1	0	*astA*	1	6	*cib*	4	0	*ibeA*	0	1
*stx1c*	1	0	*irp2*	3	3	*iha*	5	1	*neuC*	0	2
*stx2d*	6	0	*fyuA*	3	3	*subA*	6	1	*efa1*	0	1
*stx2a*	2	0	*gad*	5	6	*espP*	4	2	*vat*	0	1
*stx2e*	4	0	*tir*	0	8	*epeA*	4	0	*cdtB*	1	0
*stx1c + stx2b*	1	0	*espB*	0	6	*nleA*	0	5	*iucC*	0	1
*stx1a + stx2a + stx2c*	1	0	*espF*	0	8	*tccP*	0	5	*sab*	1	0
*eae*	0	8	*espA*	0	8	*toxB*	0	2	*celb*	2	0
*ehxA*	6	5	*espJ*	0	4	*perA*	0	2	*ireA*	2	0
*terC*	16	8	*afaD*	0	2	*etpD*	0	4	*kpsE*	2	0
*ompT*	14	5	*nleB*	0	8	*katP*	0	3	*mchB*	1	0
*chuA*	5	3	*cia*	7	2	*cea*	4	2	*mchC*	1	0
*eilA*	5	0	*cif*	0	5	*sepA*	1	0	*mchF*	1	0
*iss*	14	6	*sta1*	2	1	*stb*	1	0	*esp1*	1	1
*traT*	16	7	*cba*	3	0	*yfcV*	0	1	*cvaC*	1	0
*hra*	7	0	*cma*	4	0	*nleC*	0	4	*senB*	1	0
*lpfA*	8	3	*air*	4	0	*usp*	0	1			

## Data Availability

Data available in a publicly accessible repository.
